# A Study on Enhanced Electrorheological Performance of Plate-like Materials via Percolation Gel-like Effect

**DOI:** 10.3390/gels9110891

**Published:** 2023-11-10

**Authors:** Suk Jekal, Minki Sa, Yeon-Ryong Chu, Chan-Gyo Kim, Jungchul Noh, Jiwon Kim, Ha-Yeong Kim, Won-Chun Oh, Zambaga Otgonbayar, Chang-Min Yoon

**Affiliations:** 1 Department of Chemical and Biological Engineering, Hanbat National University, Daejeon 34158, Republic of Korea; 2 McKetta Department of Chemical Engineering and Texas Material Institute, The University of Texas at Austin, Austin, TX 78712, USA; 3 Department of Advanced Materials Science and Engineering, Hanseo University, Seosan-Si 31962, Republic of Korea

**Keywords:** electrorheological fluids, percolation, gel-like state, plate-like, ball milling

## Abstract

The use of plate-like materials to induce a percolation gel-like effect in electrorheological (ER) fluids is sparsely documented. Hence, we dispersed plate-like materials, namely natural mica, synthetic mica, and glass, as well as their pulverized particles, in various concentrations in silicone oil to form ER fluids. Subsequently, the rheological properties of the fluids were evaluated and compared to identify the threshold concentration for percolating a gel-like state. The shear stress and viscoelastic moduli under zero-field conditions confirmed that plate-like materials can be used to induce percolation gel-like effects in ER fluids. This is because of the high aspect ratio of the materials, which enhances their physical stability. In practical ER investigations, ER fluids based on synthetic mica (30.0 wt%) showed the highest yield stress of 516.2 Pa under an electric field strength of 3.0 kV mm^−1^. This was attributed to the formation of large-cluster networks and additional polarization induced by the ions. This study provides a practical approach for developing a new type of gel-like ER fluid.

## 1. Introduction

Electrorheological (ER) fluids, such as suspensions of polarizable materials in an insulating medium, contain smart materials that exhibit dramatic changes under an external electric (*E*) field [1,2]. They have received wide attention owing to their unique properties, including viscosity, storage and loss moduli, and elasticity [3,4]. Polarizable particles in an ER fluid form chains along the direction of the *E* field, significantly increasing the viscosity, and the fluid changes from liquid to a solid-like state rapidly, and vice versa [5,6]. The medium returns to its normal liquid form when the *E* field is removed, rendering ER fluids valuable for various applications, including clutch systems, haptic devices, and dampers [7,8,9]. Drastic changes in the properties of ER fluids, such as the shear stress and viscosity, can be used to evaluate the ER performance of the fluids under different *E* field strengths [5,10]. The shear stress reflects the resistance of a fluid to flow or deformation, whereas the viscosity indicates its thickness or resistance to shear stress.

ER performance, which is often evaluated in terms of shear stress and viscosity, can be influenced by various factors such as the geometric property, concentration, and polarizability of the material [11,12]. Geometric properties of the particles, including the morphology, aspect ratio, and shape, can significantly impact the responsiveness of the particles to an applied *E* field, affecting the overall performance of the ER fluid [13,14]. The polarizability and degree of polarization of an ER fluid play a crucial role in determining the dielectric constant and conductivity, particle alignment, and transient stress response of the fluid [15,16]. The concentration of ER fluids can affect the rate and extent of their property changes in response to an applied *E* field [17]. As the concentration of an ER fluid reaches its threshold or critical point, large clusters are randomly occupied or connected in the fluid from one end to another, providing a conductive pathway without an *E* field [18]. The threshold concentration can be expressed in terms of the “percolation point”. This is because when the concentration of ER fluids increases and exceeds the threshold point, the viscoelastic properties of the ER fluids increase, forming percolating gel-like states in the ER fluids [19].

Gelation is related to the formation of structures within a system. Gelation is a process in which particles cross-link to form a large chain network structure, producing a “Gel” [20]. Theoretically, a gel is a semi-solid substance that has both solid and liquid properties, with viscous and elastic characteristics [21]. The application of an *E* field can induce a gel state in ER fluids by physically linking polarizable materials through viscosity changes [22]. This change is primarily characterized by increased order and decreased mobility within the fluid [22]. The physical linkage of polarizable materials depends on the type and aspect ratio of the material [23]. The aspect ratio can considerably influence the modulus and network structure of the material during gelation. ER materials with high aspect ratios are promising candidates for realizing interfacial polarization because of their superior flow resistance, mechanical stability, geometrical properties, and dielectric properties with short relaxation times [13]. Materials with a high aspect ratio and composed of rod- and plate-type materials can be used to attain a percolating gel-like state in ER applications [24,25]. Among them, the plate-type materials exhibit a better ER performance than rod-type materials because the former has a balanced aspect ratio, which enhances their physical stability via the epigrammatic overlapping of the materials [26]. In addition, it is preferable to align plate-like materials in the direction of the applied electric field to generate a solid-like phase in the materials.

Plate-type materials are classified into three types: natural mica (NM), synthetic mica (SM), and glass (GL). They are composed of silicon-based materials such as SiO_2_ and SiO_x_ [27]. NM is a 2D material consisting of atomically thin layers of silicate minerals containing Al, K, and other elements [28]. By contrast, SM, known as fluorophlogopite mica, is a laboratory-made mica composed of fluorine-substituted minerals, such as Mg–Al silicate sheets that are weakly bound together by K [29]. Additionally, these two types of mica differ in color and texture, with SM being brighter and smoother than NM. SM contains polarization-inducing ions, whereas NM does not [30]. Furthermore, SM has a smooth edge, high durability, and high chemical and physical stability. Mica minerals such as muscovite, phlogopite, and biotite can be mixed with insulating oils and additives in specific proportions to produce novel colorful ER fluids [31]. GL is a brittle, hard, and amorphous substance which is typically transparent or translucent [32]. It is prepared by fusing sand with soda (Na_2_CO_3_) and lime (CaO), followed by rapid cooling [33]. Laboratory-made GL has exceptional physical properties such as high durability, thermal conductivity, and chemical stability [34]. Combinations of the above materials with other substances are widely used for various applications; however, few studies have investigated the dispersal of these materials in the pure state for ER fluid applications.

In this study, we used three different plate-type materials—NM, SM, and GL—and dispersed them in silicone oil for producing ER fluids. The weight concentration of the materials in the oil varied from 10.0 to 30.0 wt%. For comparison, the three plate-type materials were pulverized via a mortar–pestle-assisted ball milling process (NM/BM24h, SM/BM24h, and GL/BM24h). Next, they were dispersed in silicone oil as well. Under zero-field conditions, percolating gel-like states were observed only for the NM-, SM-, and GL-based ER fluids owing to their structural advantages. Furthermore, the SM-based ER fluids (30.0 wt%) exhibited the highest yield stress of 516.2 Pa under the application of *E* field strength of 3.0 kV mm^−1^. This is attributed to the formation of large clusters under the percolating gel-like states as well as the presence of ions facilitating high polarization. The results of this study suggest new possibilities for developing high-performance gel-like ER fluids based on plate-type materials.

## 2. Results and Discussion

### 2.1. Characterization of NM/BM24h, SM/BM24h, and GL/BM24h Materials

**Figure 1:**
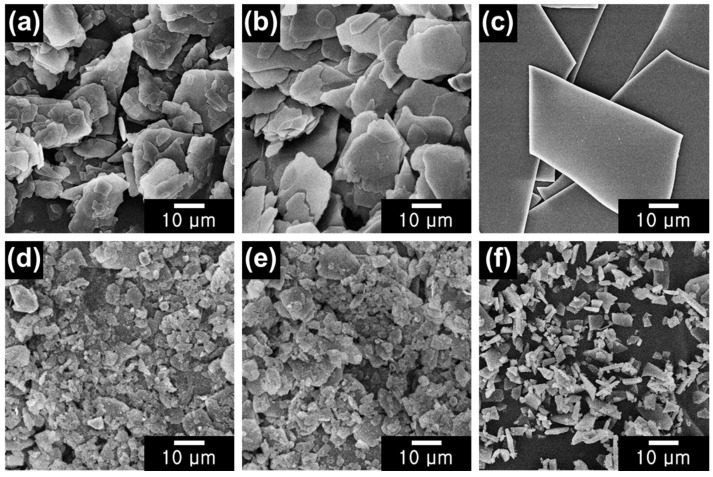
FE-SEM images of (**a**) NM, (**b**) SM, (**c**) GL, (**d**) NM/BM24h, (**e**) SM/BM24h, and (**f**) GL-BM24h.

Figure 1 shows the scanning electron microscopy (SEM) images of NM, SM, GL, NM/BM24h, SM/BM24h, and GL/BM24h. As shown in Figure 1a,b, NM and SM have smooth surfaces, with thin layers of irregularly stacked mica. SM has a smoother edge than NM, creating a smoother stacking profile in the former. NM and SM have an average particle size of ca. 25 μm, with a thicknesses of approximately ca. 0.3 μm. As can be observed from Figure 1c, GL has an average particle size of ca. 70 μm. The average particle sizes of NM/BM24h, SM/BM24h, and GL/BM24h are 5.5, 5.0, and 5.2 μm, respectively, indicating that the plate-type raw materials are completely pulverized into small pieces after ball milling.

Table 1 lists the elemental compositions of the materials, as evaluated using energy-dispersive X-ray spectroscopy (EDS). All materials primarily consist of K, Al, and Si. SM has a higher atomic percentage of Si than NM. By contrast, compared to SM, NM has higher percentages of K and Al owing to different formation conditions. Unlike mica, GL is composed of only Si without other impurities. Similar results were observed for NM/BM24h, SM/BM24h, and GL/BM24h, indicating that no other compounds or impurities were introduced during the ball milling process. Morphology and elemental composition analyses confirmed that the materials underwent a complete structural transformation during ball milling but maintained their chemical composition.

**Figure 2:**
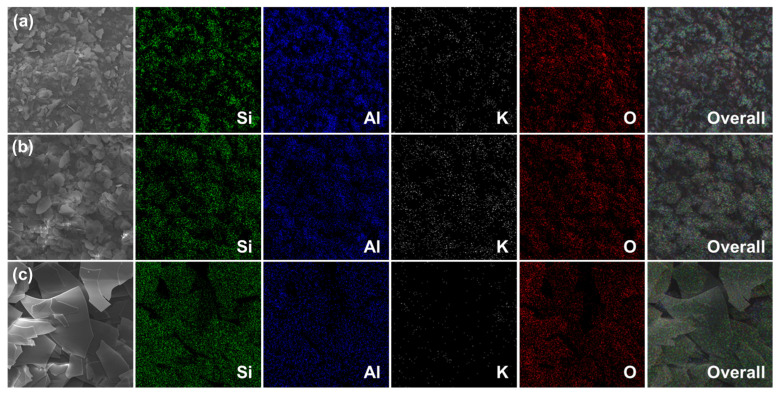
SEM and corresponding elemental mapping images of (**a**) NM, (**b**) SM, and (**c**) GL (detected elements are Si (green), Al (blue), K (white), and O (red)).

The SEM elemental mapping analysis was performed to identify traces of the primary elements present in plate-type NM, SM, and GL, as shown in Figure 2. In the mapping images, Si (green), Al (blue), K (white), and O (red) were detected in all of the plate-type materials. The elemental mapping results revealed that oxygen was present in all samples, in contrast to the EDS elemental composition results. The atomic percentages of each element were obtained to further support the elemental mapping analysis, as listed in Table 2. Noticeably, oxygen showed a higher atomic percentage than Al, Si, and K due to the analytical limitations of the EDS method in detecting light elements, which leads to an overestimation of the presence of oxygen. The atomic percentage of Si was presently higher than that of Al and K in the plate-type GL, compared with NM and SM, which indicated that the GL primarily consisted of SiO_2_. In summary, the elemental mapping images confirmed the uniform distribution of elements in all plate-type materials.

**Figure 3:**
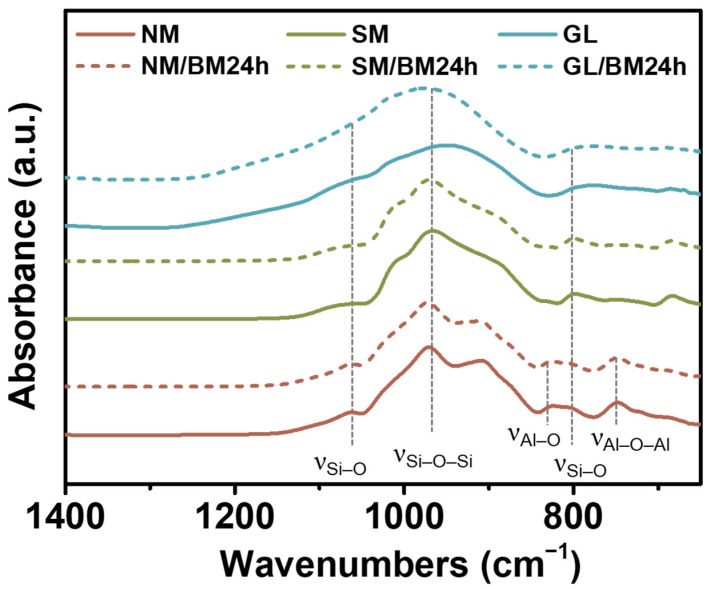
FT-IR spectra of NM, SM, GL, NM/BM24h, SM/BM24h, and GL/BM24h.

Figure 3 shows the FT-IR spectra of all the specimens. NM, SM, and GL exhibit absorption peaks at 805, 973, and 1062 cm^−1^, which are ascribed to the symmetric vibrations of the Si–O bonds, asymmetric stretching vibrations of the Si–O–Si bonds, and vibrations of the Si–O bonds, respectively [35,36,37]. In addition, NM exhibits peaks at 745 and 825 cm^−1^, which are attributed to Al–O–Al and Al–O stretching vibrations, respectively [38,39]. These peaks are produced by the high percentage of Al in NM. However, no other peak was observed for GL, which confirms the dominant composition of Si and O. The FT-IR spectra of the ball-milled samples exhibited the same absorption peaks, indicating that the molecular bonding characteristics of the plate-type materials were maintained even after pulverization.

Table 3 lists the ionic concentrations of K^+^, Na^+^, and Ca^2+^ ions in the plate-type and ball-milled materials. SM exhibits optimum concentrations of K^+^, Na^+^, and Ca^2+^ ions, whereas NM exhibits the presence of only K^+^. GL exhibits the presence of Na^+^ and Ca^2+^ ions. These ions were derived from the materials used during the preparation of the glass (sand, soda, and lime) [32]. Identical results were observed for NM/BM24h, SM/BM24h, and GL/BM24h, with little differences in the concentrations.

### 2.2. Concentration Dependence of the ER Fluids at Zero-Field Condition

**Figure 4:**
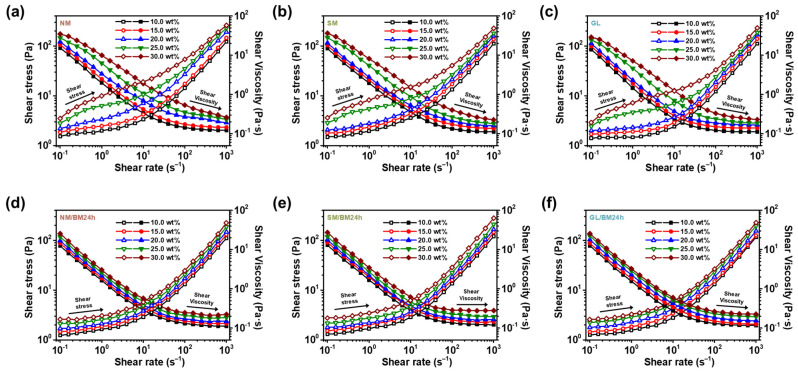
The zero-field shear stress (open symbol) and viscosity (solid symbol) curves of (**a**) NM-, (**b**) SM-, (**c**) GL-, (**d**) NM/BM24h-, (**e**) SM/BM24h-, and (**f**) GL/BM24h-based ER fluids at different concentrations ranging 10.0–30.0 wt% as a function of shear rate.

The extent of percolation and gelation in the NM-, SM-, GL-, NM/BM24h-, SM/BM24h-, and GL/BM24h-based ER fluids was evaluated in terms of rheological properties, such as shear stress and viscoelastic modulus, under zero-field conditions. The ER fluid concentrations were controlled from 10.0 to 30.0 wt% in order to investigate their effects on the rheological properties of the system. Figure 4 shows the shear stress of the materials for various concentrations. The NM-, SM-, and GL-based ER fluids exhibit dilatant behavior in the low-shear-rate regions at a concentration of 10.0, 15.0, and 20.0 wt%, respectively [40]. This behavior is maintained with increasing shear rates. However, the materials exhibit a typical Newtonian behavior in which the fluids exhibit ideal viscous strains after passing the critical shear rate (γ_c_) [41]. At a concentration of 25.0 and 30.0 wt%, the materials exhibit pseudo-plastic or gel-like behavior before the critical shear-rate regions [42]. This can be attributed to the formation of large clusters at concentrations exceeding the percolation point, which provides additional stress and enhances the physical properties and flow resistance of the plate-type materials [43]. As evident from the shear stress vs. shear rate curves, percolation gel-like effects were induced in the ER fluids at concentrations between 20.0 and 25.0 wt%. This phenomenon is not observed in the NM/BM24h-, SM/BM24h-, and GL/BM24h-based ER fluids, which indicates that the fluids did not exhibit pseudo-plastic behavior in the concentration range of 10.0–30.0 wt%, confirming the absence of a percolation point. These results further confirm that the percolation gel-like effect can be observed only in ER fluids with plate-type materials by varying their concentrations under zero-field conditions.

**Figure 5:**
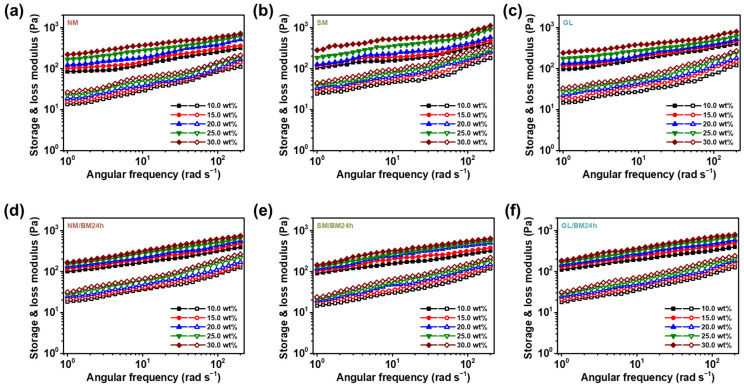
The storage modulus (closed symbol) and loss modulus (open symbol) of (**a**) NM-, (**b**) SM-, (**c**) GL-, (**d**) NM/BM24h-, (**e**) SM/BM24h-, and (**f**) GL/BM24h-based ER fluids (concentrations from 10.0 to 30.0 wt%) as a function of angular frequency.

Figure 5 shows the storage (G′) and loss (G″) moduli for the materials. For all materials, the storage modulus is higher than the loss modulus in all angular-frequency regions. Generally, the G′/G″ ratio is used to establish the state of fluids. Fluids in liquid-like and gel-like states are characterized by G′ < G″ and G′ > G″, respectively [44]. The evaluated moduli of all ER fluids revealed that all of them exhibited gel-like behavior, with G′ and G″ both increasing slightly as the angular frequency increased, further reinforcing the gel-like properties of these fluids.

**Figure 6:**
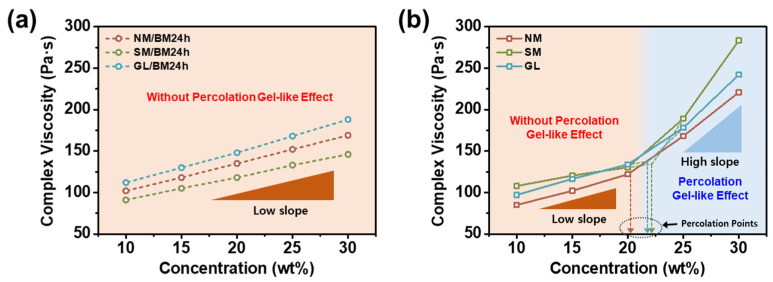
Complex viscosities of (**a**) NM/BM24h-, SM/BM24h-, and GL/BM24h-based ER fluids and (**b**) NM-, SM-, and GL-based ER fluids as a function of weight concentration in silicone oil at a constant angular frequency (1.0 rad s^−1^).

Figure 6 shows the complex viscosities of the sample as a function of concentration. As shown in Figure 6a, the complex viscosity of NM/BM24h-, SM/BM24h-, and GL/BM24h-based ER fluids linearly increases with concentration; however, the rate of viscosity increase remains constant until 30.0 wt%. By contrast, a critical concentration or threshold percolation point is observed for the NM-, SM-, and GL-based ER fluids (Figure 6b). Above the critical concentration, the slope of the complex viscosity becomes steeper, suggesting the onset of percolation and gel-like state [45]. The exact percolation points of the NM-, SM-, and GL-based ER fluids were calculated to be 20.2, 22.3, and 21.8 wt%, respectively. These findings indicate that NM/BM24h-, SM/BM24h-, and GL/BM24h-based ER fluids do not have a threshold percolation point, in contrast to NM-, SM-, and GL-based ER fluids. This is because of the physical linking of polarizable plate-type materials, which can generate network structures with large clusters in the ER fluids.

### 2.3. ER Activities of NM-, SM-, and GL-Based ER Fluids

**Figure 7:**
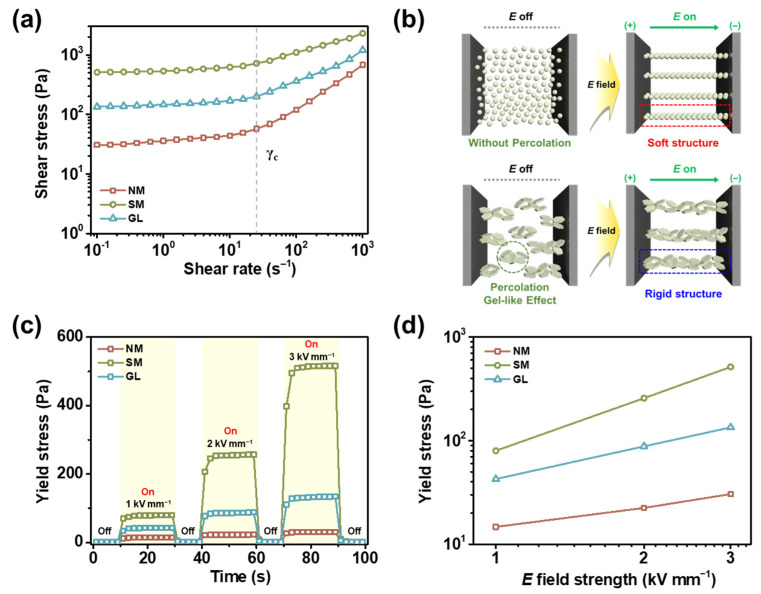
(**a**) Shear stress curves of NM-, SM-, and GL-based ER fluids (30.0 wt% in silicone oil) as a function of the shear rate under an *E* field strength of 3.0 kV mm^−1^. (**b**) Tentative mechanism of the differences in ER performance for ER fluids based on SM/BM24h (**top**) and SM (**bottom**). (**c**) *E* field on–off tests and (**d**) yield stresses of NM-, SM-, and GL-based ER fluids at various *E* field strengths ranging from 1.0 to 3.0 kV mm^−1^ with a fixed shear rate of 0.1 s^−1^.

Following the evaluation of the rheological properties under zero-field conditions, we applied various *E* fields to investigate the behavior of ER fluids consisting of plate-like materials by adjusting parameters such as the shear rate, *E* field strength, and on–off states. The concentration of the ER fluid was maintained at 30.0 wt% and silicone oil was used as the insulating medium. The shear stress of the ER fluids was determined by changing the shear rate under an *E* field strength of 3.0 kV mm^−1^. The corresponding curves are shown in Figure 7a. All plate-type materials exhibited shear stresses under an *E* field strength of 3.0 kV mm^−1^ [46]. NM-, SM-, and GL-based ER fluids exhibited a shear stress of ca. 30.5, 516.2, and 134.2 Pa, respectively. The performances of ER fluids can be distinguished when they are subjected to different shear rates. The ER fluids displayed Bingham plastic behavior at low shear rates, indicating that the electrostatic forces between the dispersed materials were strongly established without being hindered by the hydrodynamic forces caused by rotational shearing [47]. By contrast, the ER fluids exhibited Newtonian fluid-like behavior at high shear rates, indicating that rotational shearing stresses weakened the rigid and firm structures of the ER fluids [48]. The SM-based ER fluid exhibited the highest ER performance due to the synergistic effect of the percolation–gelation transition and additional polarization by K^+^, Na^+^, and Ca^2+^ ions in the material [31]. The GL-based ER fluids exhibited a shear stress between those of SM- and NM-based ER fluids owing to their ion concentration and composition [49]. Compared to laboratory-made plate materials, NM-based ER fluids exhibited the lowest ER performance because they were primarily composed of metal oxides. Figure 7b illustrates the mechanism underlying the formation of rigid structures in the SM/BM24h- and SM-based ER fluids with and without an *E* field. The small particles were evenly spread without clusters in the SM/BM24h-based ER fluids, even at high concentrations. By contrast, the SM particles dispersed in the medium agglomerated when the concentration exceeded the threshold percolation point, creating a conductive pathway without an *E* field. Upon application of the *E* field, the dispersed materials in the SM/BM24h-based ER fluids formed fibril-like structures. In the SM-based ER fluid, fibril-like structures were added to existing large clusters, thereby enhancing its ER performance. In addition, the ions contained in SM were aligned through dipole polarization, forming a more rigid structure [50,51].

The stabilities and reversibilities of the NM-, SM-, and GL-based ER fluids were demonstrated using an *E* field on–off test, as shown in Figure 7c. In the absence of an applied *E* field, all the fluids exhibited low ER performance. However, when exposed to *E* field strengths of 1.0, 2.0, and 3.0 kV mm^−1^, the yield stress increased rapidly and the behavior remained stable. The yield stresses of the ER fluids followed the order NM < GL < SM. The yield stresses immediately returned to their initial values when the *E* field was reduced, suggesting that they were reversible [52,53]. Figure 7d shows the yield stresses obtained at various *E* field strengths. Up to 3.0 kV mm^−1^, stable yield stresses are obtained without any electrical shorts, confirming the stability of the ER fluids.

**Figure 8:**
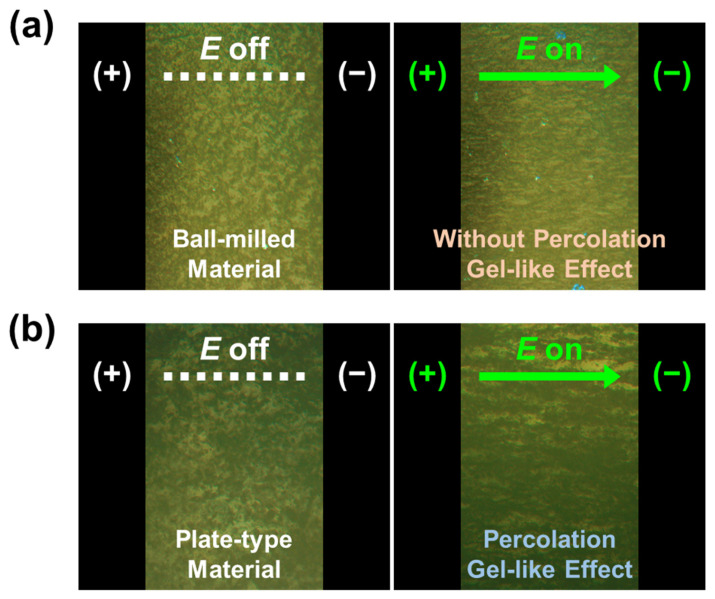
Optical microscope images for (**a**) SM/BM24h- and (**b**) SM-based ER fluids with the application of *E* field strength of 2.0 kV mm^−1^.

An ER fluid requires a real-time response analysis to ensure effective device operation. The response time of ER fluids typically falls within the millisecond range. The real-time responses of the SM/BM24h- and SM-based ER fluids (30.0 wt%) were observed using an optical microscope, as shown in Figure 8. The particles in SM/BM24h-based ER fluids were evenly distributed in the medium. Under an *E* field strength of 2.0 kV mm^−1^, the dispersed materials formed fibril-like structures rapidly. By contrast, the SM-based ER fluid was randomly distributed between the electrodes and large network structures were observed owing to the percolation gel-like effect, even in the absence of an *E* field. With the application of 2.0 kV mm^−1^ *E* field strength, additional fibril-like structures were observed with the existing percolated structure, forming more rigid structures. Real-time observations using optical microscopy confirmed the high mechanical stability of plate-type SM materials in gel-like ER fluids.

## 3. Conclusions

Plate-type materials such as NM, SM, and glass were ball-milled for 24 h. The materials underwent structural transformation but retained their chemical properties. The plate-type and ball-milled samples were dispersed in ER fluids at concentrations ranging from 10.0 to 30.0 wt%. The shear stresses and viscoelastic moduli of the materials under zero-field conditions confirmed that only ER fluids composed of plate-type materials exhibited threshold percolation points owing to their physical properties such as mechanical stability and flow resistance. The NM-, SM-, and GL-based ER fluids (30.0 wt%) exhibited yield stresses of approximately 30.5, 516.2, and 134.2 Pa, respectively. The SM-based ER fluids exhibited the highest ER performance, owing to the percolation gel-like effect and additional ionic polarization. The proposed gel-like ER fluids are promising candidates for fluids that require high ER performance.

## 4. Materials and Methods

### 4.1. Materials

NM, SM, and GL were obtained from CQV (Jincheon, Republic of Korea). Silicone oil (100 cSt) was purchased from Merck KGaA (Darmstadt, Germany). All the chemicals were used as received without any additional treatment.

### 4.2. Preparation of NM/BM24h, SM/BM24h, and GL/BM24h

NM, SM, and GL were ground using a mortar and pestle for 1 h. The ground samples were then ball-milled for 24 h (GLBM-G, Global Lab, Siheung, Republic of Korea) using zirconium oxide balls of different sizes (1 mm, 2 mm, and 5 mm). The rotational speed of the ball mill was set at 250 rpm. Subsequently, the pulverized samples (NM/BM24h, SM/BM24h, and GL/BM24h) were collected by separating the zirconium oxide balls.

### 4.3. Characterization

The morphologies and elemental compositions of NM, SM, GL, NM/BM24h, SM/BM24h, and GL/BM24h were examined using a field-emission scanning electron microscope (FE-SEM; Hitachi S-4800, Tokyo, Japan) equipped with an energy-dispersive spectrometer (EDS; HORIBA EX-250, Kyoto, Japan). The molecular composition of the samples was examined using a Fourier-transform infrared (FT-IR) spectrometer (Thermo Fisher Scientific Nicolet iS10, Waltham, MA, USA) with respect to the wavenumbers of functional groups. Ion concentrations were determined using an ion chromatographer (Metrohm 930 Compact IC Flex, Herisau, Switzerland).

### 4.4. Investigation of ER Properties

NM, SM, GL, NM/BM24h, SM/BM24h, and GL/BM24h were dispersed in silicone oil without additives, followed by magnetic stirring for 6 h. The concentrations of the samples varied from 10.0 to 30.0 wt%. The ER properties of the samples were assessed using a modular rheometer (Anton Parr MCR 102, Graz, Austria) with a concentric cylinder geometry (C-CC17/T200/SS). The rheometer was equipped with a cup and high-voltage generator (Fug Elektronik HCP 14-12500, Schechen, Germany). A distance of 1.0 mm was set between the cup and cylinders without any obstructions. The ER fluids were stirred under homogeneous conditions and then poured into the cup. The fluids were pre-sheared for 5 min at a shear rate of 10.0 s^−1^ for ensuring a uniform distribution. The viscoelastic characteristics including shear stress, on–off *E* fields, and dynamic modulus were obtained under different *E* field strengths ranging from 0 to 3.0 kV mm^−1^ at 25 °C. Angular frequency sweep tests were conducted to evaluate the viscoelastic properties of NM-, SM-, GL, NM/BM24h-, SM/BM24h-, and GL/BM24h-based ER fluids as a function of angular frequenc*ies* ranging from 1.0 to 200 rad s^−1^ without an *E* field.

The complex viscosity of the ER fluids was determined at a constant angular frequency of 1.0 rad s^−1^, with the concentration of ER fluids fixed at a value between 10.0% and 30.0%. The following equations were employed to evaluate the complex viscosity and modulus [54,55]:

G* = *G*′ + *iG*″, (1)

η* = G*/*ω*, (2)

where *G*′, *G*″, G*, η*, and *ω* are the storage modulus, loss modulus, complex modulus, complex viscosity, and angular frequency, respectively. The complex modulus was calculated using the storage and loss moduli, and the resulting data were utilized to plot the complex viscosity with respect to concentration. The values obtained were used to compare the percolation points at different weight concentrations. The percolation points were evaluated using the double tangent line crossing method.

## Figures and Tables

**Table 1:** Elemental compositions of NM, SM, GL, NM/BM24h, SM/BM24h, and GL/BM24h *^a^*.

Samples	Elemental Composition (wt%)		
	Si	Al	K
NM	44.6	40.6	14.8
SM	69.5	18.3	12.2
GL	100.0	- *^b^*	-
NM/BM24h	42.4	41.2	16.4
SM/BM24h	70.5	18.6	10.9
GL/BM24h	100.0	-	-

*^a^* Elemental composition of samples were obtained using EDS mode in FE-SEM (accelerating voltage: 10.0 kV, beam current: 10.0 μA). *^b^* Elemental composition was not detected in the sample.

**Table 2:** Atomic percentages of Si, Al, K, and O contained in plate-type materials *^a^*.

Samples	Atomic Percentage (%)			
	Si	Al	K	O
NM	16.7	13.0	4.2	66.1
SM	19.5	8.6	6.2	65.7
GL	34.6	2.6	1.8	61.0

*^a^* Atomic percentages of elements were evaluated from EDS elemental mapping (accelerating voltage: 20.0 kV).

**Table 3:** Concentration of K^+^, Na^+^, and Ca^2+^ ions in NM, SM, GL, NM/BM24h, SM/BM24h, and GL/BM24h *^a^*.

Samples	Ionic Concentrations (ppm)		
	K^+^	Na^+^	Ca^2+^
NM	3.5	- *^b^*	-
SM	9.7	20.6	12.5
GL	-	5.4	2.6
NM/BM24h	3.3	-	-
SM/BM24h	9.2	19.5	13.1
GL/BM24h	-	4.8	3.0

*^a^* Ionic concentrations were measured using Metrohm 930 compact IC Flex system. *^b^* Ionic concentrations was not detected in the sample.

## Data Availability

Data are contained within the article.
